# Passive Monitoring of Short-Acting Beta-Agonist Use via Digital Platform in Patients With Chronic Obstructive Pulmonary Disease: Quality Improvement Retrospective Analysis

**DOI:** 10.2196/13286

**Published:** 2019-10-23

**Authors:** Jessica Chen, Leanne Kaye, Michael Tuffli, Meredith A Barrett, Shelanda Jones-Ford, Tina Shenouda, Rahul Gondalia, Kelly Henderson, Veronica Combs, David Van Sickle, David A Stempel

**Affiliations:** 1 ChenMed Miami, FL United States; 2 Propeller Health San Francisco, CA United States; 3 JenCare Senior Medical Center Louisville, KY United States; 4 Content Strategy Solutions Louisville, KY United States; 5 Propeller Health Madison, WI United States

**Keywords:** chronic obstructive pulmonary disease, telemedicine, quality improvement, feasibility, nebulizers and vaporizers, health services

## Abstract

**Background:**

Digital health programs assist patients with chronic obstructive pulmonary disease (COPD) to better manage their disease. Technological and adoption barriers have been perceived as a limitation.

**Objective:**

The aim of the research was to evaluate a digital quality improvement pilot in Medicare-eligible patients with COPD.

**Methods:**

COPD patients were enrolled in a digital platform to help manage their medications and symptoms as part of their routine clinical care. Patients were provided with electronic medication monitors (EMMs) to monitor short-acting beta-agonist (SABA) use passively and a smartphone app to track use trends and receive feedback. Providers also had access to data collected via a secure website and were sent email notifications if a patient had a significant change in their prescribed inhaler use. Providers then determined if follow-up was needed. Change in SABA use and feasibility outcomes were evaluated at 3, 6, and 12 months.

**Results:**

A total of 190 patients enrolled in the pilot. At 3, 6, and 12 months, patients recorded significant reductions in daily and nighttime SABA use and increases in SABA-free days (all *P*<.001). Patient engagement, as measured by the ratio of daily active use to monthly active use, was >90% at both 6 and 12 months. Retention at 6 months was 81% (154/190). Providers were sent on average two email notifications per patient during the 12-month program.

**Conclusions:**

A digital health program integrated as part of standard clinical practice was feasible and had low provider burden. The pilot demonstrated significant reduction in SABA use and increased SABA-free days among Medicare-eligible COPD patients. Further, patients readily adopted the digital platform and demonstrated strong engagement and retention rates at 6 and 12 months.

## Introduction

Chronic obstructive pulmonary disease (COPD) affects more than 65 million adults worldwide and is expected to become the third leading cause of death by 2030 [1]. COPD is associated with progressive loss of lung function and has significant impacts on both physical and mental well-being [2].

The use of inhaled long-acting muscarinic antagonist, long-acting beta-agonists alone or in combination with a long-acting muscarinic antagonists, or an inhaled corticosteroid–long-acting beta-agonist combination has been associated with improved symptom control and decreases in exacerbations associated with COPD [3]. Short-acting beta-agonists (SABA) are prescribed to manage acute symptoms. Knowledge of actual SABA use and its signal of increased risk may be used to adjust medication regimens and identify worsening symptoms and determine risk or occurrence of an exacerbation [4-6]. Historically, information on patient use of SABA has been self-reported, leading to challenges of recall bias through incorrect recollection or fabrication [7-9].

To address the burden and morbidity of COPD [10,11], novel approaches for disease management are warranted [12]. Web-based self-management, smartphone apps, electronic medication monitors, and text-based interventions have been used to track, automate, and provide real-time feedback on medication use and clinical status. These digital health interventions have demonstrated success for chronic respiratory diseases including asthma and COPD [13-17]; however, these programs may be limited by nonengagement and attrition [18,19].

Emerging research suggests that digital health programs integrating providers [20] or human coaches may improve clinical outcomes, rates of engagement, and retention [21,22]. However, there has been little effort to engage COPD patients in these programs due to perceived challenges with patient age and comfort with technology [18,23,24], as well as perceived increases in provider burden. This analysis aimed to determine the feasibility of integrating a digital quality improvement pilot in a clinical setting to improve outcomes in a COPD Medicare population.

## Methods

### Recruitment and Eligibility

Patients were recruited from three JenCare Senior Medical Center clinics in Louisville, Kentucky, from December 2015 to December 2016. JenCare clinics are managed care facilities that provide high-touch care to low- to moderate-income Medicare-eligible patients. Inclusion criteria were kept broad to include as many patients as possible in the quality improvement pilot. Patients with a physician diagnosis of COPD, using a sensor-compatible SABA medication, and fluent in English or Spanish were eligible for enrollment. An in-person enrollment process was led by clinical team members and two respiratory therapists. Eligible patients were required to accept Propeller Heath’s Terms of Use [25] prior to participation, which permits the use of aggregated, de-identified data in analyses. This analysis was determined to be exempt by the Copernicus Independent Review Board.

### Study Design

**Figure 1:**
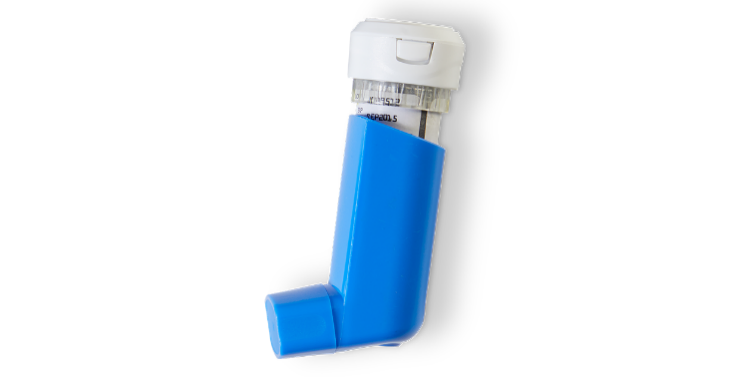
Electronic medication monitor that captures inhaler medication use.

Patients were provided with an electronic medication monitor (EMM; Propeller Health) that attached to their compatible SABA (Figure 1). The EMM was used to objectively monitor the date and time of SABA use. The EMM was paired via Bluetooth to a smartphone, which transmitted the information to Health Insurance Portability and Accountability Act–compliant servers. Patients without smartphones received a wireless hub to transmit EMM data passively. Lost or malfunctioning EMMs and hubs were replaced.

The EMM provided is part of a US Food and Drug Administration–cleared digital health platform comprising a mobile app, Web dashboards, and short message service and email modalities [13,14,26]. Patients had access to a Web or mobile platform that promoted self-management with information about their SABA use trends, potential environmental triggers, and guidelines-based education [3]. Further, because this was a quality improvement pilot, there was no formal retention plan such that patients were not required to have a final visit or last sensor sync. No incentives were provided.

### Health Care Providers and Electronic Notifications

Patients authorized their health care providers to view collected data and summary reports through a secure Web dashboard. This information could be used to inform clinical treatment such as medication adjustments or early intervention at the sign of increasing SABA use. In addition, at-risk notifications were sent to providers if a patient was considered at increased risk for an exacerbation (defined as use of ≥10 SABA puffs in a 24-hour period or SABA use above the patient’s personal baseline level for two or more consecutive days). Based on these notifications, providers could determine appropriate follow-up with a patient via telephone or an in-person visit as needed. A respiratory therapist assisted patients with technical questions by phone and in person.

### Feasibility Outcomes

We evaluated patient retention at 3, 6, and 12 months (defined as an EMM sync with at least 3, 6, and 12-month data points, respectively). Syncs demonstrate that the EMM is capable of collecting and transmitting data via a smartphone or hub. While syncs do not require actuation of the medication, they do require that the patient maintain the digital platform. For example, patients must ensure the hub remains charged and connected to an outlet and that the EMM is transferred to any refilled inhaler prescriptions. As such, to evaluate patient engagement, we examined the proportion of days that a sync was recorded at 3, 6, and 12 months. We also compared the percentage of daily active users with the percentage of monthly active users to determine level of daily engagement. Finally, we assessed provider notification burden by averaging the number of at-risk notifications sent to the provider per patient over the 12-month program.

### Short-Acting Beta-Agonist Use

Change in mean SABA use and percentage of SABA-free days was assessed at 3, 6, and 12 months. The date, time, and number of SABA puffs were recorded as mean puffs per day. Nighttime SABA use was calculated as a subcategory of daily use, defined as use between 10:00 pm and 6:00 am. Percentage of SABA-free days were calculated as the percentage of days (unique 24-hour periods) in which a patient did not use their SABA inhaler.

### Statistical Analyses

Descriptive statistics were completed for the feasibility outcomes at 3, 6, and 12 months. SABA use outcome analyses compared the change in the mean number of SABA uses per day, mean number of nighttime SABA uses, and percentage of SABA-free days from baseline to specified end points. The baseline period was defined as week 1 (days 1-7), with day 1 as the first EMM sync. We evaluated outcomes at baseline and compared against three end points: 3 months (week 13), 6 months (week 26), and 12 months (week 52) among those patients with sufficient follow-up. To account for potential biases due to attrition during the 12-month study period, we calculated stabilized inverse probability of attrition weights for each participant with 3 months of data, conditional on age, race, gender, season of enrollment, smartphone or hub use, baseline SABA use, and syncing using logistic regression [27]. We then estimated changes in SABA use parameters from baseline to 3, 6, and 9 months using attrition-weighted longitudinal, mixed-effects linear models adjusting for age, race, gender, and season of enrollment. All analyses were conducted in R version 3.4 (R Foundation for Statistical Computing).

## Results

### Population

A total of 190 patients enrolled in the digital quality improvement pilot. Patients were primarily white (99/190, 52.1%) or African American (85/190, 44.7%), female (124/190, 65.3%), and over age 60 years (153/190, 80.6%) with a mean age of 68.0 (SD 9.2) years (Table 1). Almost all patients transmitted data using the wireless hub device (184/190, 96.8%).

**Table 1:** Patient demographics (n=190).

Characteristics		n (%)
**Age in years**		
	40-49	1 (0.5)
	50-59	36 (18.9)
	60-69	67 (35.3)
	70 and older	86 (45.3)
Gender, female		124 (65.3)
**Race**		
	White	99 (52.1)
	African American	85 (44.7)
	Other	4 (2.1)
	Unknown	2 (1.1)
**Device type**		
	Hub	184 (96.8)
	Smartphone	6 (3.2)

### Feasibility Outcomes

Patient retention was 90.5% (172/190) at 3 months, 81.0% (154/190) at 6 months, and 63.1% (120/190) at 12 months (Table 2). There were no significant differences in age, race, gender, baseline SABA use, or sync history between those patients who completed 12 months and those who did not (Table 3). The percentage of daily active users to monthly active users remained above 90% for all three end points. Similarly, the mean proportion of days patients synced at 3, 6, and 12 months was 91%, 90%, and 88%, respectively. Providers were sent 397 at-risk notifications over 12 months, a mean of 2.1 notifications per patient.

**Table 2:** Feasibility and process outcomes.

Characteristics		Value
**Patient retention, n (%)**		
	3 months	172 (90.5)
	6 months	154 (81.0)
	12 months	120 (63.1)
**Patient engagement (proportion of days with at least one sync), %**		
	3 months	91
	6 months	90
	12 months	88
	Daily active user/monthly active user	90.5
Provider at-risk alerts, n (mean per patient)		397 (2.1)

**Table 3:** Comparison of baseline characteristics between patients with complete and noncomplete follow-up at 12 months.

Characteristic		Patients who completed 12 months (n=120)	Patients who did not complete 12 months (n=70)	*P* value
**Age, n (%)**		—	—	.38
	40-49	1 (1)	0 (0)	—
	50-59	18 (15)	17 (24)	—
	60-69	44 (37)	24 (34)	—
	70 and older	57 (48)	29 (41)	—
**Race, n (%)**		—	—	.35
	White	61 (51)	38 (54)	—
	African American	56 (47)	29 (41)	—
	Other	3 (3)	1 (1)	—
	Unknown	0 (0)	2 (3)	—
Female, n (%)		82 (68)	42 (60)	.31
SABA^a^ at baseline, puffs/day, mean (SD)		3.1 (2.3)	4.0 (1.6)	.18
Days with at least one sync at baseline, %		94	88	.09

^a^SABA: short-acting beta-agonist.

### Change in Short-Acting Beta-Agonist Use

**Figure 2:**
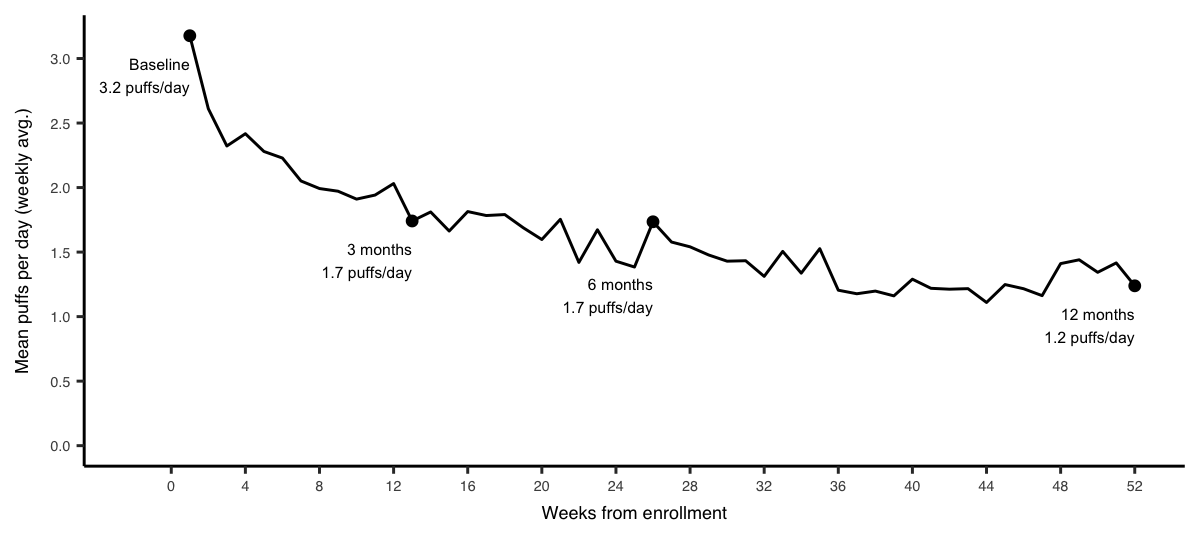
Attrition-weighted and adjusted mean daily short-acting beta-agonist use at 3, 6, and 12 months.

**Figure 3:**
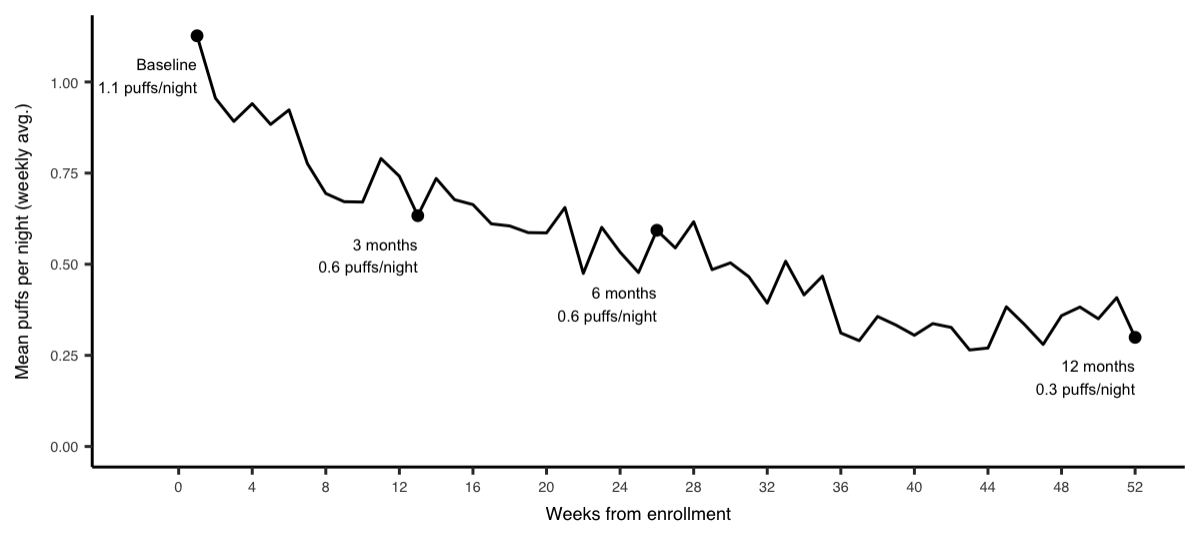
Attrition-weighted and adjusted nighttime short-acting beta-agonist use at 3, 6, and 12 months.

**Figure 4:**
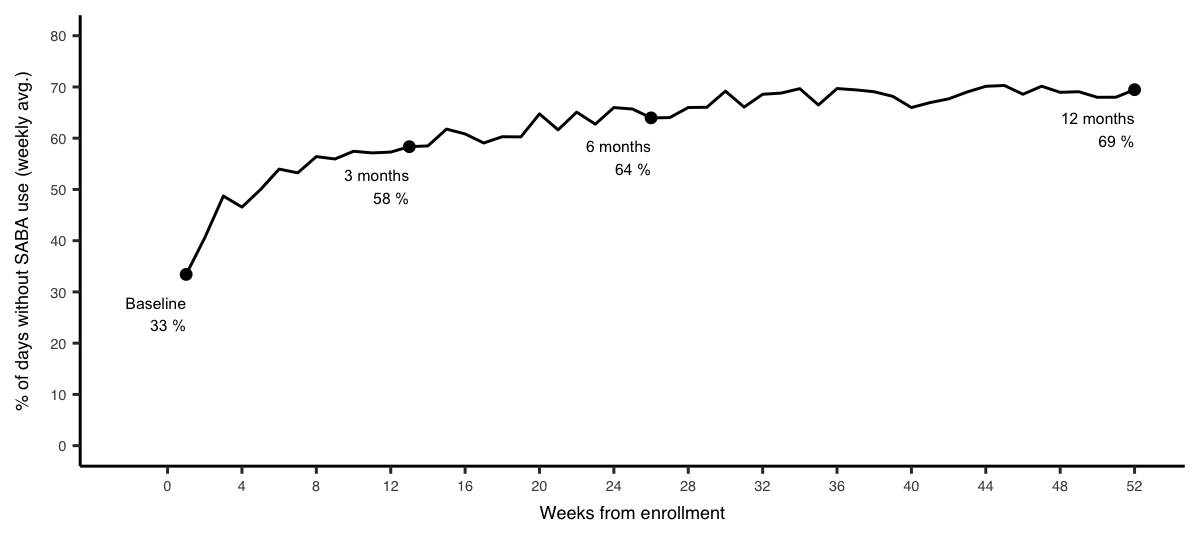
Attrition-weighted and adjusted percent of short-acting beta-agonist–free days at 3, 6, and 12 months.

In crude analyses, decreases in mean SABA use and nighttime SABA use and increases in SABA-free days were observed over the study period in patients with 3, 6, and 12 months of follow-up (Multimedia Appendix 1). After accounting for potential biases due to attrition and adjusting for potential confounders, mean SABA use, nighttime SABA use, and SABA-free days were 3.2 puffs per day, 1.1 puffs per day, and 33%, respectively. SABA use decreased on average by 1.4 (95% CI –1.6 to –1.2), 1.6 (95% CI –1.7 to–1.2), and 1.9 (95% CI –2.1 to –1.7) puffs per day, from baseline to 3, 6, and 12 months, respectively (Figure 2). Similarly, nighttime SABA use decreased by 0.5 (95% CI –0.6 to –0.4), 0.5 (95% CI –0.7 to –0.4), and 0.8 (95% CI –0.9 to –0.7) and the weekly proportion of SABA-free days increased by 25% (95% CI 22 to 28), 31% (95% CI 27 to 34), and 36% (95% CI 33 to 39) points from baseline to 3, 6, and 12 months, respectively (Figures 3 and 4).

## Discussion

### Principal Findings

This study evaluated the feasibility of a clinically integrated digital quality improvement pilot in a Medicare-eligible COPD population. Patients demonstrated significant improvements from baseline to 3, 6, and 12 months for reduced daily and nighttime SABA use and an increased percentage of SABA-free days. Further, this study demonstrated that a digital health program was feasible in this COPD population.

Providers had access to the patient’s clinical status and real-time use of SABA. Providers received at-risk notifications at the patient level to alert them to increased SABA use and provide the opportunity for earlier intervention. While we did not formally record changes to medication regimens, other studies have shown that real-time identification of increased SABA use enables more timely provider intervention and may result in lower exacerbation rates and acute health care utilization including hospitalizations and emergency department visits [4,5]. Additional randomized controlled studies are needed to confirm this effect.

### Comparison With Prior Work

Previous studies have demonstrated that engagement rates for digital health applications may peak around 3 months and decline rapidly thereafter [28,29], with approximately 10% of participants engaging at 6 months [30]. In this study, we saw 81% retention and 90% of days with a sync at 6 months. The three recruitment centers are characterized by high-touch, engaged providers. It is possible that the clinical improvement and engagement in this study may be explained in part by the frequency and quality of the provider engagement. For example, a previous meta-analysis exploring the impact of provider communication on patient engagement found a positive correlation between provider feedback and medication adherence rates [31]. Additional research shows that active provider engagement may improve retention rates [32,33]. Patient-provider data sharing and transparency may also improve outcomes and engagement through shared decision-making [15,34].

Digital health interventions may also increase provider burden [23]. However, this analysis suggests that passively tracking medication use and integrating this information into a comprehensive clinical care model may result in stronger patient engagement without excessive provider burden. For example, only 2.1 at-risk notifications were sent on average per patient throughout the 12-month study period. Future studies should address provider burden and satisfaction in a more robust manner.

EMMs may reduce patient burden. Measures of medication use in clinical studies frequently rely on self-report, placing the burden on patients and increasing the likelihood of recall bias [28]. By objectively and passively collecting medication use data and transmitting it to the provider, both patient burden and reporting accuracy may be improved.

### Limitations

This study is limited, in part, by its design as a single-arm study and regression to the mean for SABA use should be considered. Further, controller medication use was not tracked due to a noncompatible formulary, limiting the opportunity for controller medication adherence promotion and possible impact on SABA use. It is also possible that patients used additional SABA inhalers without attaching an EMM. Measurement of engagement and clinical outcomes was limited and future programs should consider more definitive measures a priori. Finally, factors beyond the EMMs and platform may have contributed to the observed outcomes, and future randomized studies are needed to confirm the findings.

### Conclusion

This quality improvement pilot demonstrates that a digital health program with integrated clinical care is feasible in a Medicare-eligible population of COPD patients. The results demonstrated strong retention rates at 12 months, with reduced daily and nighttime SABA use and more SABA-free days. With the increasing medical resources being used for COPD treatment [10], novel approaches for disease management are warranted [12].

## Appendix

Multimedia Appendix 1 Changes in short-acting beta-agonist use.

## Abbreviations

COPD: chronic obstructive pulmonary disease
EMM: electronic medication monitor
SABA: short-acting beta-agonist
