# Supplementary material for: Passive Monitoring of Short-Acting Beta-Agonist Use via Digital Platform in Patients With Chronic Obstructive Pulmonary Disease: Quality Improvement Retrospective Analysis
Source: JMIR Form Res. 2019 Oct 23;3(4):e13286. doi: 10.2196/13286 (PMC7010108; doi:10.2196/13286)
Supplement: Multimedia Appendix 1 [file formative_v3i4e13286_app1.pdf]

Multimedia Appendix 1. Changes in short-acting beta-agonist use.

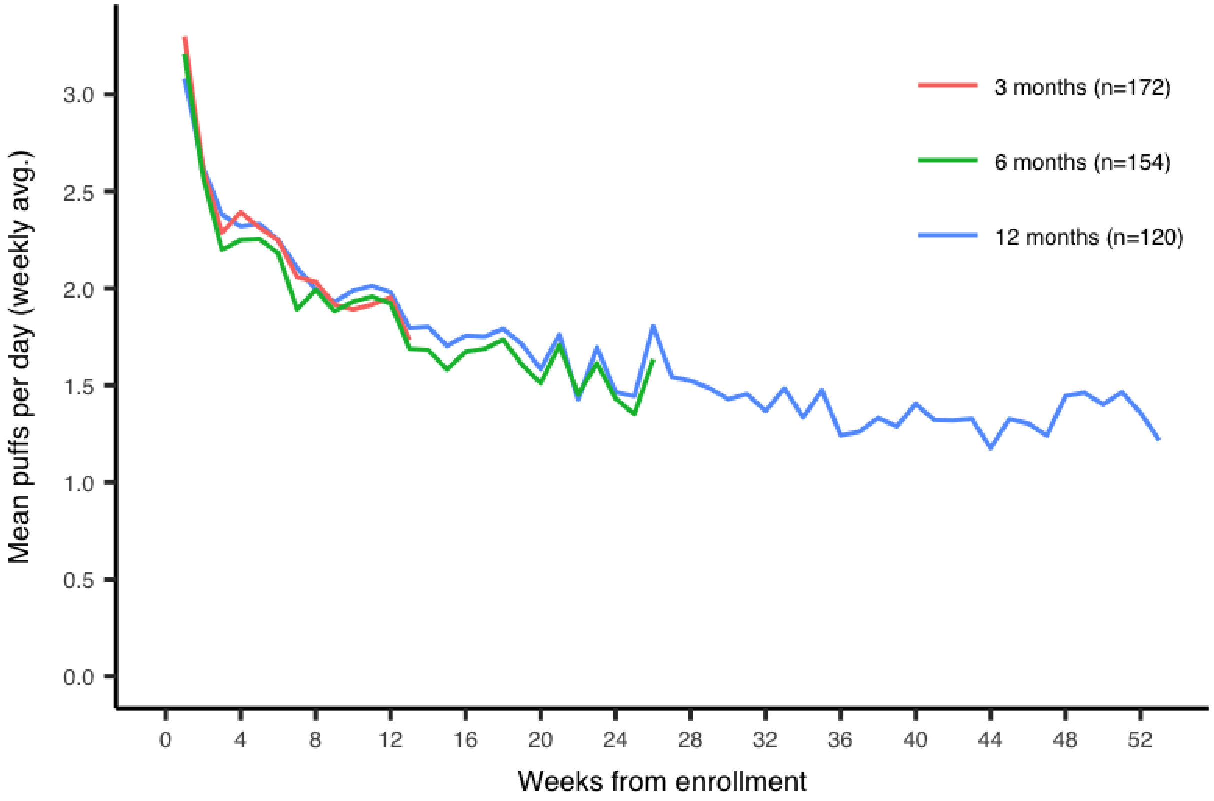

Figure S1. Mean daily SABA use at 3, 6, and 12 months

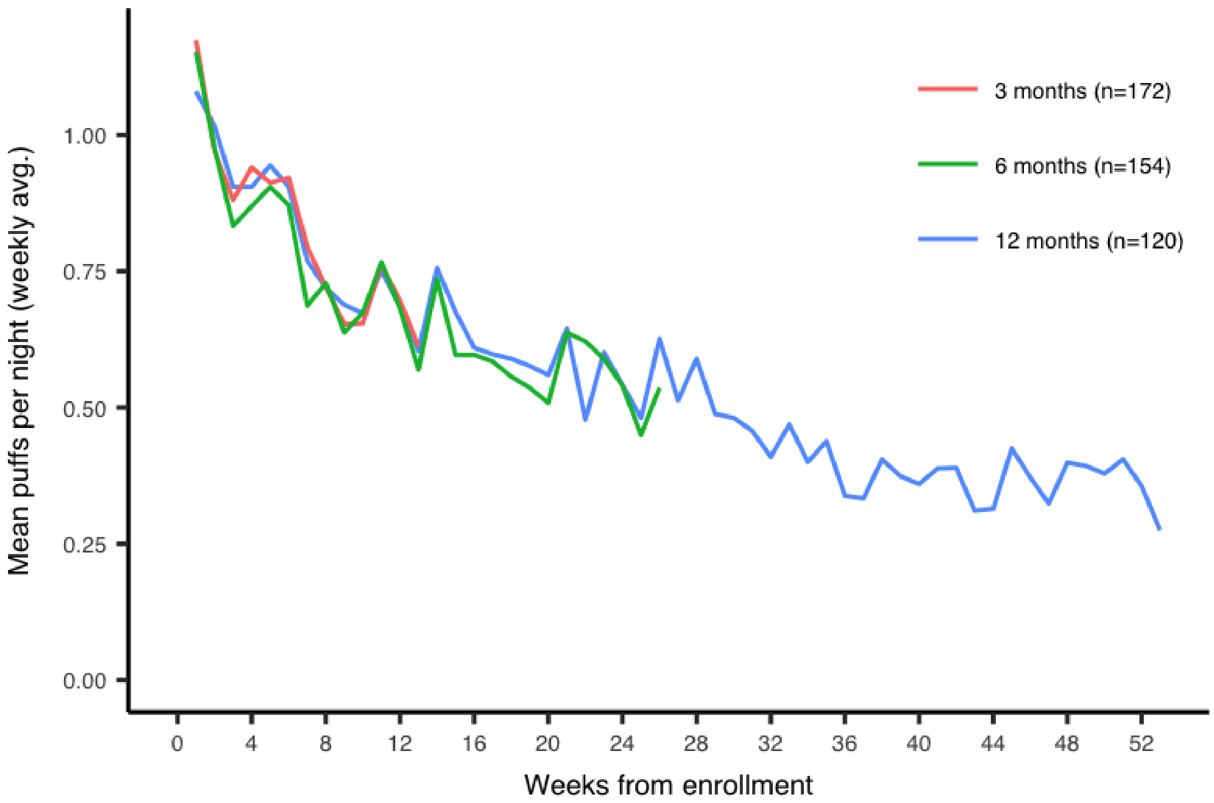

Figure S2. Nighttime SABA use at 3, 6, and 12 months

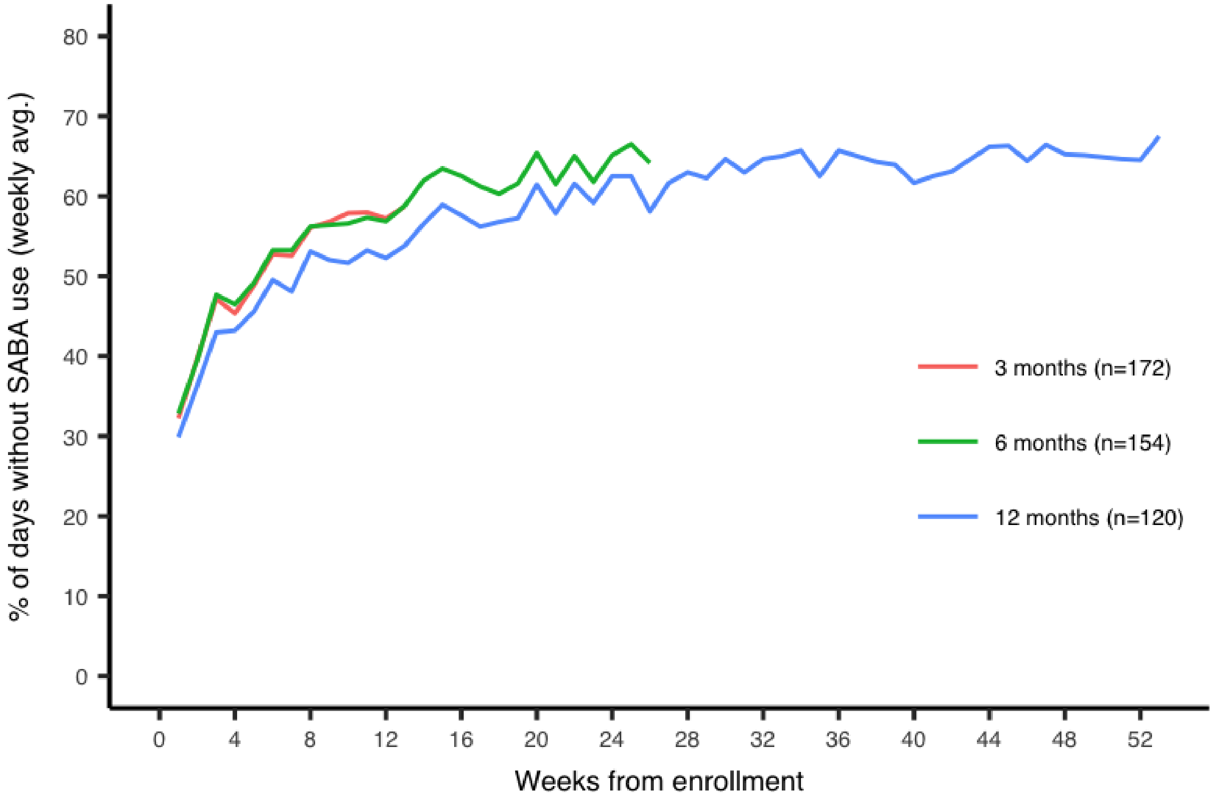

Figure S3. Percent of SABA-free days at 3, 6, and 12 months
